# Potential Roles of Exosomal MicroRNAs as Diagnostic Biomarkers and Therapeutic Application in Alzheimer's Disease

**DOI:** 10.1155/2017/7027380

**Published:** 2017-07-09

**Authors:** Jian-jiao Chen, Bin Zhao, Jie Zhao, Shao Li

**Affiliations:** ^1^Department of Physiology, Liaoning Provincial Key Laboratory of Cerebral Diseases, Dalian Medical University, Dalian City, Liaoning Province 116044, China; ^2^Department of General Surgery, Subei People's Hospital of Jiangsu Province, Yangzhou City, Jiangsu Province 225000, China; ^3^Technology Centre of Target-Based Nature Products for Prevention and Treatment of Aging-Related Neurodegeneration, Dalian Medical University, Dalian, Liaoning, China

## Abstract

Exosomes are bilipid layer-enclosed vesicles derived from endosomes and are released from neural cells. They contain a diversity of proteins, mRNAs, and microRNAs (miRNAs) that are delivered to neighboring cells and/or are transported to distant sites. miRNAs released from exosomes appear to be associated with multiple neurodegenerative conditions linking to Alzheimer's disease (AD) which is marked by hyperphosphorylated tau proteins and accumulation of A*β* plaques. Exciting findings reveal that miRNAs released from exosomes modulate the expression and function of amyloid precursor proteins (APP) and tau proteins. These open up the possibility that dysfunctional exosomal miRNAs may influence AD progression. In addition, it has been confirmed that the interaction between miRNAs released by exosomes and Toll-like receptors (TLR) initiates inflammation. In exosome support-deprived neurons, exosomal miRNAs may regulate neuroplasticity to relieve neurological damage. In this review, we summarize the literature on the function of exosomal miRNAs in AD pathology, the potential of these miRNAs as diagnostic biomarkers in AD, and the use of exosomes in the delivery of miRNAs which may lead to major advances in the field of macromolecular drug delivery.

## 1. Introduction

Alzheimer's disease (AD) is a progressive neurodegenerative disease which is marked by aggregation of *β*-amyloid (A*β*) plaques and formation of neurofibrillary tangles (NFTs) derived from hyperphosphorylated tau proteins whose overall outcome results in neural dysfunction [1]. Majority of AD patients do not show any symptoms during the first few years (3.3 years) before the onset of dementia, at which time memory impairment might have reached its threshold [2]. Therefore, early diagnosis before the clinical phase is quite essential and could be effective for therapeutic prevention.

There are many exciting findings that have emerged in the field of extracellular vesicles during the last few years. These findings that show that application of exosomal contents which causes a change in neurological damage condition provides a bright future for the diagnosis of neurodegenerative disease [3–6]. In the central nervous system (CNS), not only neurons and astrocytes secrete exosomes but microglia and oligodendrocytes also secrete exosomes into the extracellular environment [7]. Exosomes have generated immense interest in the field of neurodegenerative disorders after their discovery as mediators delivering important proteins [8], short interfering RNA (siRNA) [9], and microRNAs (miRNAs) [3–6] in intercellular communication. Altered microRNA profiles in cerebrospinal fluid/blood exosomes associated with neurodegenerative disorders reveal new biomarkers in the diagnosis of AD [3–6], and the fact that exosome is able to deliver siRNA introduces a therapeutic potential to AD [3, 6, 10].

Just like small noncoding RNAs, miRNAs have functional effects on posttranscriptional regulation of gene expression and could serve as novel diagnostic biomarkers for AD [11]. Profound works have been done to identify freed miRNAs in AD with anticipation of introducing reliable diagnostic biomarkers and to clarify molecular pathways that are associated with the formation of dysfunctional tau and A*β* aggregates [12].

Exosomal miRNAs and their pattern of transfer deserve more attention in order to establish their emerging roles and potential applications in AD. In this review, we summarize the existing knowledge of exosomal miRNAs and their involvement in AD, emphasizing their potential to be used as diagnostic biomarkers during the preclinical phase of AD as well as the possibility of them being used to regulate neurodegeneration.

## 2. Exosomes—Natural miRNA Carriers

### 2.1. Biogenesis Pathway, Release, and Uptake of Exosomal miRNA

Exosomes are small-sized (between 30 and 100 nm) spherical vesicles with an endosome-derived membrane enriched with lipids such as cholesterol, ceramide, and sphingolipids. They contain cell state-specific loads of protein, mRNA, and miRNA [13]. Some studies have shown that specific miRNAs have been involved in AD pathogenesis where the miRNAs bind directly to 3′-UTR of mRNA and repress protein synthesis at the posttranscriptional level causing them to exert regulative effects [14–16]. Apart from these classical pathways (gap junction channels, apoptosis bodies, and synaptic cleft), exosomes are proposed to carry functional miRNAs coupled to the enzyme Argonaute 2 and migrate into the surrounding cells [17]. Transporting miRNAs are very secured in exosomes and retain their functional activity in target cells.

#### 2.1.1. Formation and Secretion of Exosomes

Exosomes are derived from intraluminal vesicles of late endosomal compartments named multivesicular bodies (MVBs). It is the specific contents and membrane molecules that are contained that define exosomes irrespective of the cell types they originated from. Established exosomes contain molecules that participate in MVB biogenesis like TSG101 and Alix. They could also contain proteins involved in signal transduction such as heat shock proteins (HSP70, HSP90), certain cytoplasmic proteins such as tubulin and actin, Annexin and Rab family proteins, tetraspanins (CD9, CD63, and CD81), and major histocompatibility complex class I (MHC-I) molecules, as well as the various transmembrane proteins [7].

The fundamental step in MVB formation involves the organization of specialized domains called tetraspanin-enriched membrane domains (TEMs). TEMs are enriched with membrane proteins that are necessary for vesicular fusion. In addition, TEMs also recruit some exosome membrane proteins which can be potential ligands for receptor-mediated internalization into the recipient cells [18]. Another specific class of membrane proteins that make up exosomes are components of endosomal sorting complex required for transport (ESCRT) machinery. These endosomal sorting complexes recognize the intraluminal vesicles which give rise to the formation of MVBs containing miRNAs. Fusion of MVBs with plasma membrane gives rise to microvesicles named exosomes which are released into the extracellular space [19]. Another important protein, Alix, is considered as a biomarker of exosome and acts together with TSG101 and CHMP4. It engages in various steps of the exosome biogenesis including cargo packaging and vesicle formation [20].

During the release of exosomes into extracellular space, any of the following could happen: (1) they may be caught by surrounding cells (or by identical cells they are derived from); (2) they may be captured by cells within distal sites; or (3) they may enter systematic circulation and be internalized into various cells [21]. Exosomes may exert its function in several distinct modes: (i) internalization into target cells and delivering their contents into these cells, (ii) attaching to the target cell's surface to trigger a second messenger signaling pathways, and (iii) releasing its contents into the extracellular space [22].

#### 2.1.2. Biogenesis and Maturation of miRNAs

miRNAs are derived from long primary transcripts that are split by a complex of protein DGCR8/Pasha and RNAse III Drosha. These are shorter hairpin structures (50–120 nt) that constitute miRNA precursor (pre-miRNA) in the nucleus. The pre-miRNA is then transported into the cellular cytoplasm, converted to a short nucleotide duplex by the RNAse III enzyme Dicer, and then conveyed to miRNA-containing RNA-induced silencing complex (miRISC). In the miRISC, the single mature miRNA, which is derived from the pre-miRNA hairpin components, combines with an Argonaute protein and then migrates to specific neuronal sites [23, 24].

**Figure 1:**
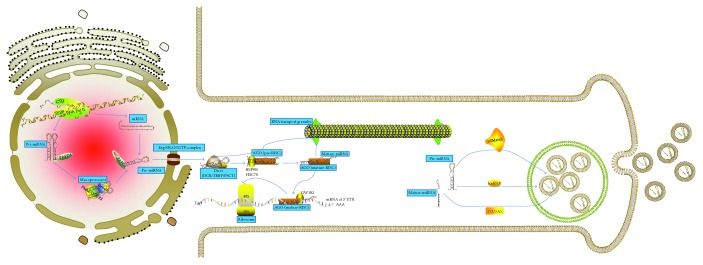
The biogenesis pathway of exosomal miRNA and composition of exosome. Canonical miRNAs are initially transcribed from long (41 kb) endogenous precursors called primary miRNAs that are driven by RNA pol II promoters and cleaved by microprocessor, a multiprotein complex formed by the RNase type III Drosha (the Drosophila homolog of RNASEN in humans) and the protein DGCR8 (Di George Syndrome Critical Region 8). A hairpin structure (now known as pre-miRNA) with 60–80 nucleotides in length, which bears a two-nucleotide overhang at the 3′ end that is a mark left by the Drosha processing, is released in this step. Within neuronal nuclei, pri- and pre-miRNA may be stabilized by 3′-terminal adenylation performed by PAPD4. After recognizing the precursors by their overhangs, GTPase-dependent Ran-Exp5 complex exports the pre-miRNAs out of the nucleus to the cytoplasm. An alternative pathway needs splicing out of the miRNAs from introns located in other genes, then further lariat processing, and finally proper folding into a pre-miRNA structure. In the cytoplasm, cleavage of the pre-miRNAs takes place once they have been loaded onto the Dicer-TRBP (TAR-RNA binding protein) complex, which removes the loop from the pre-miRNA to produce a dsRNA duplex that contains both the mature miRNA (or leader strand) and the so-called passenger strand. The Dicer-TRBP complex rapidly transfers the duplex to the miRISC (miRNA-RNAi-induced silencing complex), which contains AGO (Argonaute proteins) as its core. The HSP90/HSC70 chaperone complex participates in the process. In the miRISC, the passenger strand is degraded by an unidentified mechanism, leaving a mature miRISC loaded with a fully mature single-stranded miRNA (19–22 nt) to bind canonically to non-fully complementary mRNA targets at their 3′ untranslated regions (UTR). Alternatively, pri-miRNAs and miRNA may be loaded with proteins of RNA transport granules. These molecules are then transported to specific neuronal compartments, where mature or precursor miRNAs are enveloped in exosomes to be released elsewhere. Mature miRNAs are sorted into exosomes via four potential mechanisms: (1) the neutral sphingomyelinase 2- (nSMase2-) dependent pathway; (2) the miRNA motif and sumoylated heterogeneous nuclear ribonucleoprotein- (hnRNP-) dependent pathway; (3) the 3′-end of the miRNA sequence-dependent pathway; and (4) the miRNA-induced silencing complex- (miRISC-) related pathway (not shown).

Alternatively, pre-miRNA and miRNA processing machinery may be collected with RNA transport granule proteins. These molecules are subsequently exported to specific neuronal sites, where either the matured or precursor miRNAs are packaged into exosomes. It must be noted that exosome as a carrier of miRNAs is novel. A schematic representation of exosomal miRNA formation is shown in Figure 1.

#### 2.1.3. The Sorting Mechanism for Exosomal miRNAs

According to recent literature, the process of loading miRNAs into exosomes follows four main proposed ways even though the exact mechanisms has not been concluded on. Potential modes include the following: (1) the neutral sphingomyelinase 2- (nSMase2-) dependent pathway: the nSMase2 increases the quantum of exosomal miRNAs. When nSMase2 expression and function is inhibited specifically, the quantity of exosomal miRNAs reduces, indicating the involvement of nSMase2 in how miRNA is packaged into exosomes [25]. (2) The sumoylated heterogeneous nuclear ribonucleoprotein- (hnRNP-) dependent pathway: GGAG motif in the 3′ portion of miRNA sequences can be recognized by sumoylated hnRNPA2B1, and once this recognition occurs, specific miRNAs are then packaged into exosomes [26]. (3) The 3′-end of the miRNA sequence-dependent pathway: Koppers-Lalic and his colleagues reported that the 3′-ends of uridylated endogenous miRNAs exist in exosomes that originate from B cells. The 3′-ends of an adenylated miRNA sequence are also present in B cells, indicating that the 3′ portion of miRNA is very vital in giving the signal to sort [27]. In addition to the above three selective modes, Guduric-Fuchs and his colleagues also revealed a possible involvement of miRISC in exosomal miRNA sorting mechanism, that is, (4) the miRNA-induced silencing complex- (miRISC-) related pathway: when AGO2 is knocked out, the main component of miRISC which prefers to bind to the 5′-end of miRNA reduces the number of privileged-transported miRNAs, such as miR-451 and miR-150, in HEK293T-derived exosomes [28]. Other findings have also confirmed the relationship and involvement of miRISC with exosomal miRNA sorting mechanism [29]. Specific sequences of miRNAs may guide their loading into exosomes, while some proteins may also contribute to the sorting of exosomal miRNAs.

### 2.2. Localization of Exosomal miRNA in Neural Compartments

Since the CNS appears to be an abundant store of miRNA expression, it is suggested that the relevance of miRNA within the CNS is determined by neural-specific miRNA epigenetic modulative effect during brain embryogenesis [30]. Microarray analyses show how the function of miRNA regulates its function epigenetically during CNS development. This shows an important aspect of neural function especially neuroplasticity [31]. A study by Zhang et al. has indicated that specific miRNAs are profusely expressed in fetal hippocampus. In their work, the turnover in miRNA complexity occurred in AD hippocampus and therefore supported the hypothesis that modifiable miRNA-mediated processing of mRNA group may result in neural dysfunction [32].

As a carrier and transporter of miRNA, exosomes are disseminated from various cells including neurons [33] and astrocytes [34]. In addition, circulating exosomes in neurodegenerative disorders have a distinct miRNA signature that may be involved in the pathology of these disorders [3, 4]. It is possible that changes in miRNA expression exert an essential but uncharacteristic role in the neurodegenerative progression. There is limited evidence supporting this in the literature available; hence, the alteration in exosomal miRNA signature could be a result of the disease pathology which needs further research to clarify.

### 2.3. Association of Exosomal miRNAs with Neurodegeneration

Exosomes are endosome-derived membrane vesicles that carry a variety of cellular proteins, mRNA and miRNA [35]. Some findings strongly support the hypothesis that exosome-mediated miRNA signature could be utilized in the diagnosis of neurodegenerative diseases. The decrease in cerebrospinal fluid-derived exosomal microRNA-193b [3] and the downregulation of plasma exosomal miR-342-3p [4] could be the signatures. Exosomal miRNAs have the potential to be a risk factor like age, sex, and apolipoprotein *ε*4 (APOE *ε*4) used for predicting AD [6].

Corresponding to these findings, some specific exosomal miRNAs exhibit sufficient function in reducing memory decline and cognitive dysfunction. Application of low-level IFN*γ* released exosomes (IFN*γ*-DC-Exos) that contained miR-219 exerts functional effects including increase in myelination, reduction of oxidative stress, and improvement of remyelination [36]. In addition, exosomes which originate from multipotent mesenchyme stromal cells (MSCs) transfer packaged miR-133b to astrocytes and neurons promoting neural plasticity and neurite remodeling [37]. Besides, studies by Xin et al. that investigated exosome-derived biomarkers in neuropsychiatric diseases proposed two agents, miR-497 and miR-29c, which are expressed profoundly in the prefrontal cortices of bipolar disorder and schizophrenia patients which provided an alternative view concerning the pathogenesis of these diseases [37]. Furthermore, it was studied that miRNAs encapsulated in exosomes in the cerebrospinal fluid (CSF) undergo temporal and spatial alteration during CNS embryogenesis and development and thus concluded that this could be of physiological importance [38]. Overall, miRNAs involved in the pathogenesis of AD deserves attention in order to explore the fluctuations in exosome expression.

## 3. Epigenetic Regulation of Exosomal MicroRNAs Associated with Alzheimer's Disease

### 3.1. Neuronal Intercellular Communication Involving Exosomal miRNA

Cells must communicate with each other through exchange of information and substances constantly in order to maintain physiological homeostasis. It is generally accepted that several communication modes exist in CNS. Among these, the outstanding mode is through signal activation initiated by neurotransmitters in synaptic transmission between axons and their terminals. A growing number of evidence demonstrate that exosomes may be one of several distinct mechanisms to guarantee various trades of cellular information and substance transfer [39]. The findings that exosome mediates cytosol transfer broaden the comprehension of cellular communication which account for additional physiological and pathophysiological processes within the brain. Recently, De Toro and his team figured out the diagnosis and therapeutic potential of exosomes in neurodegenerative disorders [7].

A new exciting pathway, exosome-mediated transfer of miRNA signal from neuron to astrocyte, has been characterized: excitatory amino acid transporter 2 (EAAT2, rodent analog GLT1) in perisynaptic astrocytes modulates direct miRNA-124a transfection mediated by exosome, and this leads to selective intensification of glutamate transporter1 (GLT1) protein (but not mRNA) expression in astrocytes [40].

While neurons transfer miRNA into astrocytes through neuronal exosomes and then significantly regulate protein expression in an indirect manner [40], dendritic cells may produce exosomes containing miRNA following acute lysolecithin-induced demyelination. The exosomes that are released integrate with beneficiary cells to supply practically miRNAs that increase baseline myelination and reduce oxidative stress [36]. In addition, conveyance of miR-133b boxed in exosome-enriched extracellular particles between MSCs and neural cells promotes neural plasticity. This enhances functional recovery of learning and memory in Alzheimer's disease [37]. In that regard, apart from conveying miRNAs as messengers in neural communication, exosomes may also modulate neural regeneration.

### 3.2. Modulation of Neurodegenerative Signaling Pathways by Exosomal miRNA

Investigation of miRNA reveals posttranscriptional modulation function as a mechanism regulated epigenetically. A considerable amount of evidence put forward by Hu et al. as well as Ismail et al. points out that miRNAs mediated by exosomes exert their effects in the recipient cell and may convert target gene expression [34, 41]. Mature miRNAs bind to complementary portions within the target mRNA sequence and cause them to degrade or repress the translation process. Numerous miRNAs can bind at the 3′-UTR of specific genes, while identical miRNAs may have several targets; miRNA networks therefore allow the coordination of gene expression [42].

Several specific miRNAs bind to complementary sites within 3′-UTR of key genes that determine the expression of amyloid precursor protein (APP) and beta-site APP-cleaving enzyme (BACE) [43, 44]. In AD brain, extracellular A*β* plaques, which ultimately lead to progressive loss of neurons, are derived from processing of APP by BACE. Significantly deregulated miRNAs targeting APP like miR-193b [3], miR-101 [45], or BACE1 like miR-29c [43, 44] influence A*β* generation in AD brain. Researchers have validated computational predictions and demonstrated that miRNAs recognize specific complementary sites within 3′-UTR of APP/BACE1 mRNA, and they pointed out a possible involvement of miRNA in AD [46].

Numerous findings in transgenic AD mice identify that some specific miRNA expression deviates from the general levels and that deregulation of these miRNAs can contribute to the accumulation of extracellular A*β* plaques in sporadic AD [47, 48]. Unusual miRNA expression is supposed to be the cause for irregular proteosomal degradation of insoluble and phosphorylated tau proteins, which are mediated by Cdk5/p25 activation [49]. Genetic ablation of Dicer in adult forebrain neurons results in hyperphosphorylation of tau causing neuronal loss in the hippocampus and ultimately impairing cognitive function [50].

Potentially, apart from degrading erroneous proteins, miRNAs transferred by exosomes may also alleviate protein scarcity via rectifying functional protein translation [51]. Association of miRNAs with Ago2 protein rectifies such biological effect on misfolded proteins and renders them functional [52]. In addition to cellular miRNAs, specific virus-encoded miRNAs may also be loaded into exosomes. Epstein-Barr virus (EBV) encoding viral miRNAs may be packaged into exosomes from infected B-cells and repress the immune-regulatory gene CXCL11 in surrounding dendritic cells derived from monocytes [53]. Besides, HIV-encoded transactivation response element (TAR) miRNA could be released via exosomes and this can affect their ability to infect recipient cells [54]. All these findings broaden our knowledge and understanding about the functions of exosomal miRNAs, and this may lead us to other emerging functions.

### 3.3. Interplay between Exosomal miRNA and Toll-Like Receptors in Neuroinflammation

Neuroinflammation is responsible for the “double-edged sword” effect in Alzheimer's disease. Future studies that will investigate the relationship between molecules associated with neurodegeneration and receptors that initiate inflammation will bring some understanding to the mechanism of neurodegeneration [55].

**Figure 2:**
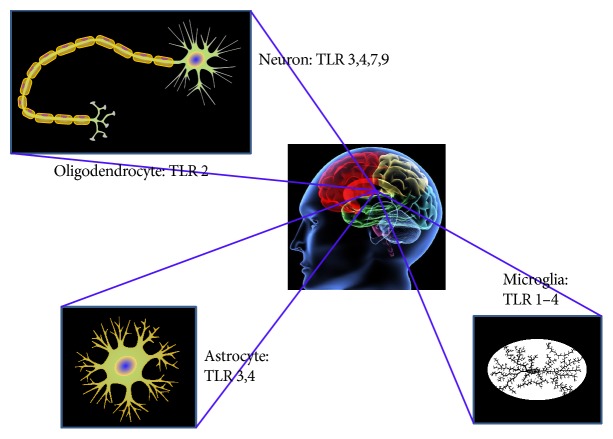
Neural cell types and their Toll-like receptor (TLR) expression. Protein profile for TLR3, 4, 7, and 9 has been reported in different neural phenotypes from humans; only TLR2 protein profile is detected in human oligodendrocytes; TLR3-4 protein accumulation is found in human astrocytes; human microglia contains TLR1–4 protein profile.

It is conjectured that miRNAs mediated by exosomes may initiate Toll-like receptor (TLR) activation under certain circumstances [56, 57]. Sohrabifar et al., in their work, found that TLRs pertain to a group of pathogen-associated molecular pattern receptors and also reported that TLR polymorphisms can be related to late-onset Alzheimer disease (LOAD) susceptibility [58]. During the last decades, TLRs have been found in various cells in the brain [59–62], as shown in Figure 2. The relationship between miRNA mediated by exosomes and TLRs was deemed important in discovering the role of exosomal miRNAs in neuroinflammation of AD.

Some findings suggest that miRNAs participate in TLR-signaling pathway at various levels, including the following: (1) targeting some components of the TLR signaling system, such as their proteins associated with signaling, regulatory molecules, and transcription factors as well as functional cytokines induced by them; (2) miRNA expression may directly be regulated by TLRs activation pathway, and (3) miRNAs may directly activate the RNA-sensing TLRs [63].

Lehmann and his group have shown that TLR7–9 recognize specific miRNAs as ligands in the CNS. For example, miRNA let-7, which is highly expressed in microglia and neurons, interacts with TLRs to regulate factors of transcription induced by TLR. Therefore, it is inferred that let-7 binds to RNA-sensing TLR7 and consequently induces neurodegeneration through neuronal TLR7 [64]. Besides, the up- and downregulation research of miR-146a brings out into the open that miR-146a may act as a potent mediator of microglial function in response to TLR2 stimulation during neurodegeneration [65]. Furthermore, in AD mouse and human brain, miR-146a localized to the hippocampal regions is full of proinflammatory cytokines in response to TLRs. These levels constitute disease severity and suggest the link between miR-146a and inflammation-induced neuropathology [66]. Zhang and his group concluded that polymorphisms in pri-miR-146a and the rare C allele of rs2910164 may also contribute to the genetic predisposition to AD by disrupting the production of miR-146a-5p which affect the expression and function of TLR2 [67]. Based on these findings, we can confirm that some specific miRNAs play a role in CNS's innate immune response and is a potential regulator modulating microglial function during neurodegeneration.

### 3.4. Regulatory Components of Exosomal miRNA in the Neuroplasticity Dynamics

Rapid input-restricted change in gene expression is an important aspect of neural plasticity that requires very complex mechanisms of regulating and transferring posttranscriptional mRNA. Small noncoding miRNA are positioned uniquely to support these functions by providing a nucleic acid-based specificity component for universal sequence-dependent RNA binding complexes. As a special cellular vehicle, exosomes loaded with specific miRNAs may benefit from neuroplasticity under adverse environmental conditions.

Redistribution of miRNA associated with rapid depolarization in neurons was observed by Goldie and his group and provided evidence that miRNAs exert regulatory effects on the dynamics of normal synaptic function. Intriguingly, they also found that a large proportion of depolarization-associated changes attributed to the release of exosomes enriched with evolved miRNAs suggesting the essential vehicular role of exosome [33]. Systemic administration of exosomes that are released from mesenchymal stromal cells (MSCs) enhance neurite remodeling, neurogenesis, and angiogenesis [68, 69], and this effect may be a result of neural plasticity enhanced by exosomal miRNAs. Exosomes derived from MSCs transfer miR-133b to astrocytes and neurons, and this subsequently increases axonal plasticity and benefits from neurite remodeling [37, 70].

Gray matter injury is firmly associated with cognitive dysfunction. It was found that cognitive decline follows with neurodegeneration from myelin loss and that age-associated deficiency due to remyelination significantly contributed to AD progression [71]. Research by Pusic et al. demonstrated that environmental enrichment with serum-derived exosomes that contained miR-219 are critical for the production of myelinated oligodendrocytes, and this can be done by reducing the expression of inhibitory differentiation regulators [72]. What is more, dendritic cells (DCs) that were stimulated with IFN*γ*-released exosomes (IFN*γ*-DC-Exos) and enriched in miRNA species reduced oxidative stress increased baseline myelination and improved remyelination following acute lysolecithin-induced demyelination [36]. Interestingly, these exosomes containing specific miRNAs that had been derived from neuronal progenitor cells promoted neuronal differentiation of MSC and thus provides potential applications for tissue regeneration [73].

These findings further support the hypothesis that exosomal miRNA could be used as neural plasticity regulator. The association of the abovementioned exosomal miRNAs with neurodegeneration are summarized in Table 1. As synaptic function is thought to be dysfunctional in neurodegenerative conditions, it is plausible that exosomal miRNA signature could be used as a potential diagnostic marker for Alzheimer's disease.

## 4. Exosomal miRNAs: Novel Insights into Biomarkers for AD

Current therapeutic activities for neurodegenerative disorders are restricted not only due to curative deficiency but also due to the limited understanding of their underlying mechanisms and also the difficulties posed in accurately diagnosing AD during the subclinical stages. Since pathological changes initiated years before the appearance of clinical symptoms, predictable biomarkers for the detection of AD are critical so much that preventative strategies could be applied to retard cognitive decline. In a neurodegenerative disorder research by Bellingham and his colleagues, a small RNA deep sequencing study demonstrated that neuronal exosomes contained a diverse range of RNA species which included retroviral RNA repeat regions, messenger RNA fragments, transfer RNA fragments, noncoding RNA, small nuclear RNA, small cytoplasmic RNA, and silencing RNA as well as known and novel candidate miRNA and that these circulating exosomes had a distinct miRNA signature that could be utilized for diagnosing neurodegenerative disorders [74].

### 4.1. Feasibility of Exosomal miRNAs as Biomarkers for AD

Recent development in molecular biology has prompted the potential of biomarker research from a notion of pathogenesis and specific incident that leads to a disorder originating from biosystem deterioration. Specific profiles of exosomal miRNAs from human biological fluids such as plasma and CSF have prompted the potential application of miRNAs as diagnostic biomarkers [3–6]. However, there are few obstacles that stand in the way, especially how specific miRNAs can pass through the blood-brain barrier (BBB), a mechanism that is unknown. The BBB serves as a barrier with specialized molecular machinery that mediate delivery of macromolecules, while making sure that viruses and bacteria fail to pass through it [75]. Under normal conditions, A*β* is mediated by low-density lipoprotein protein receptor that transports it across the BBB, suggesting the possibility of other neurodegenerative biomarkers to cross this barrier. Several presumable mechanisms by which miRNAs can cross the BBB have been put forward. In substance, it is a general consensus that some pathological conditions in neurological disorders may cause perforations of BBB which consequently leads to the release of cellular components [75]. In a study, the postmortem brains of late-stage AD patients reported that neurodegenerative conditions disrupt BBB vascular basement membrane [76]. Inspiringly, exosomes are proposed to function as transporters of miRNA that traverse the endothelial cellular layers of the BBB and this could facilitate communication between the brain and distant organs via biological fluids [77]. It has been proposed that the mechanism that enables exosomes to cross endothelial cells of the BBB can be by a receptor-mediated endocytosis [78]. In this regard, miRNAs mediated by exosomes may serve as a class of biomarkers in neurodegenerative disorders.

Alterations that occur in a biosystem often affect substances within exosomes released by neural cells, and these enlighten the possibility of exosomal miRNAs as potential biomarkers for diverse pathological states wherein deregulated miRNAs may be used for early diagnosis. In a study, exosomal miRNAs obtained from postmortem prefrontal cortices of subjects who had been diagnosed with schizophrenia and bipolar disorders were varied significantly from matched controls [79]. It was also reported that schizophrenic patients exhibited elevated levels of exosomal miR-497, while elevated levels of exosomal miR-29c were also found in patients with bipolar disorder. Furthermore, researches identifying neurodegeneration-associated exosomal miRNAs in the CSF may verify the utility of exosomes to serve as biomarker carriers for neurological diseases. Furthermore, miR-193b has been shown to be present in the hippocampus of AD mice. It has been demonstrated that overexpression of miR-193b could suppress the expression of APP. This shows that miR-193b may get involved in the neurodegenerative process, and therefore, exosomal miR-193b has the potential to be a unique biomarker for AD [3]. It is unlikely that a single specific miRNA could be used as AD-associated signature. In researches looking for biomarkers that correlate with diagnosis of AD, a 7-miRNA AD-associated signature was obtained in a machine learning model for predicting AD status of individuals with 83–89% accuracy [4]. A similar study also selected an AD-specific 16-miRNA signature, along with established risk factors contributed to predicting AD [6]. Intriguingly, in Parkinson's disease (PD) research by Gui et al., microRNA profiling strategy for exosomal miRNAs isolated from CSF was suggested to serve for differential diagnosis of PD with AD [5]. The purpose of exosomal miRNA biomarker researches is to be able predict future cognitive decline in asymptomatic individuals and the progression of the disease in patients with early dementia.

### 4.2. Appropriate Biological Fluids for Detecting Exosomal miRNAs

Analysis of biological fluids for detecting brain-associated miRNAs has been focused on CSF and blood so far. As biological fluid yielded in the choroid plexus, CSF circulates through the inner ventricular system, crosses the BBB, and is integrated into the bloodstream. On the basis of these characteristics, CSF may serve as the appropriate source containing biomarkers for AD [3, 5]. However, owing to its invasive process of sample collection, biomarkers associated with blood for AD testing may be much more widely applicable and acceptable. Identifiable miRNAs as biomarkers in blood exhibit an added advantage for early clinical diagnosis. Nevertheless, scanty researches identify miRNA as biomarkers for AD patients using blood samples. In a research that assessed the value of exosomal miRNAs as biomarkers for AD, the P3 fraction of plasma, isolated by differential centrifugation and enriched in exosomes, revealed a profile of miRNA changes occurring in AD [4]. In another research, exosomes isolated from serum proved to be highly enriched in miRNA and an AD-specific 16-miRNA signature represented a vital step towards developing a cost-effective, noninvasive, and low-risk diagnostic application. Furthermore, it is feasible to isolate exosomes from plasma and serum by differential ultracentrifugation to profile-specific exosomal miRNAs. Generally, urine is collected noninvasively to obtain urine cellular sediment from low-speed centrifugation to analyze miRNAs implicated in prostate and bladder cancers [80]. However, analysis of urine cell sediments may not be appropriate for neurodegenerative diseases in the sense that it is enriched with cell debris that originate from the hematologic and renal epithelial as well as certain microorganisms. With appropriate samples, advances to improve sensitivity and accuracy in detecting diagnostic biomarkers would be valuable in investigating the application of exosomal miRNAs as novel biomarkers for neurodegenerative disorders.

### 4.3. Innovative Approach for Analysis of Exosomal miRNA

Over the last decades, new approaches to obtain large quantities of exosomes have been applied in trials including ultrafiltration, and the methods for attempting to acquire pure exosomes have been developed as utilization of monoclonal antibodies targeted membranous marker proteins of exosomes. Furthermore, exosomes have been classified according to various characteristics such as their density, morphology, size, expression of marker proteins, and intracellular origin [7].

Studies of CSF from AD patients have applied the use of miRNA microarrays, multiplex miRNA qPCR assay, or target candidate miRNA approaches to identify differentially expressed miRNA [81]. Recently, the progress in deep-sequencing technology has presented a realizable approach to detect deregulated exosomal miRNAs in neurodegenerative diseases [4, 6]. Deep-sequencing method permits exhaustive analyses of numerous sequence data, initiating revolutionary accelerated research compared to traditional methods. This has shown to be an effective approach to identifying accurate encoded information from nucleotide fragments [82]. Benefits of deep sequencing rely on their capability to be simultaneously operated on a large quantity of independent sequencing events. Due to the sensitivity and accuracy of deep sequencing, it is able to identify genetic modifications, particularly deregulated miRNA, in neurodegenerative disease [82].

## 5. Synthetic miRNA Delivered by Exosomes: A Potential Therapy for AD

Previous reports have demonstrated that dysfunction of miRNAs is involved in the pathogenesis of human AD and that the therapeutic potential of miRNAs could guide us towards the development of new therapeutic strategies in neurodegenerative disorder. In a study, upregulating miR-188-3p in the hippocampus suppressed BACE1 expression and A*β* formation and this prevented deteriorations in long-term potentiation, spatial learning, and memory in the hippocampus [32]. It is unlikely that miR-188-3p is unique in its ability to regulate A*β* formation, but the role of miR-188-3p is of critical interest, given that its recognition element in the BACE1 3′-UTR is extraordinarily conserved. Apart from the accumulation of A*β*, another neuropathological hallmark of AD, neurofibrillary tangles (NFTs) result from tauopathy which may also be regulated by miRNA as well. For example, miR-219 binds directly to the 3′-UTR of the tau mRNA and represses tau synthesis at the posttranscriptional level. Modification of the silencing mechanism of tau by miR-219 that is perturbed during neurofibrillary degeneration suggested that developing therapies for tauopathies is possible [14]. In this case, favorable natural transport of nanovesicles represent another major barrier to avoid or alleviate immunogenicity of the miRNA or its delivery vehicle, especially if repeated doses are needed to treat degenerative diseases.

Recent researches have revealed that exosomes mediate horizontal delivery of distinct RNAs in intercellular communication. The utilization of exosomes for transport of certain therapeutic nucleic acids to recipient cells is considered as a potential approach for AD [51]. Involvement of exosomal miRNAs with pathogenesis of neurodegenerative disorders urges researchers to investigate their therapeutic potential too.

The capacity of exosomes to deliver functional miRNA pushes the hope of replacing the already known virus-based gene therapy with this [83]. When this is compared to current RNA interference (RNAi) approaches which includes utilization of viral and synthetic delivery systems such as liposomes and endogenous exosomes, the body's own intercellular carrier may internalize into recipient cells without evoking any immune responses. This therefore assures that an advancement in the field of drug delivery is near possibility [7].

**Figure 3:**
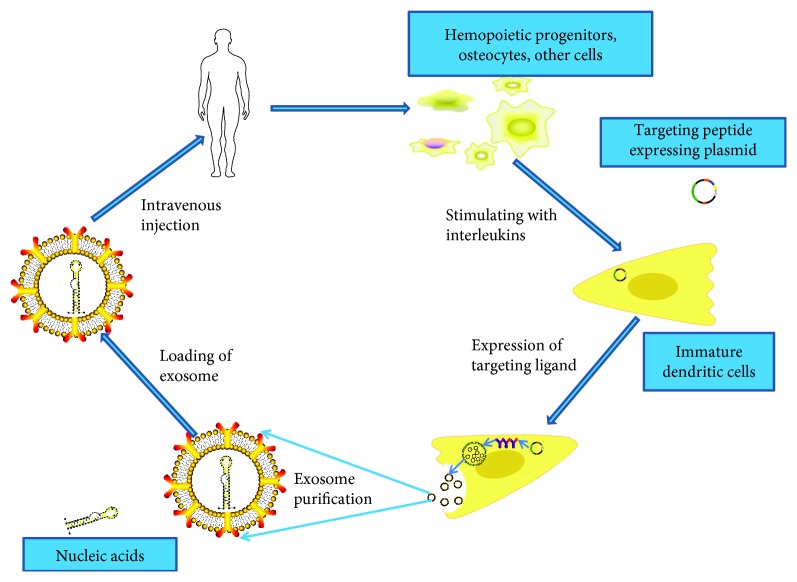
Schematic representation of production, harvest, and readministration of engineering modified exosomes for gene delivery. To acquire enough immunologically inert exosomes, harvest cells like hemopoietic progenitors and osteocytes are used as the source cell. As immature dendritic cells produce a lot of exosomes devoid of T-cell activators such as MHC-II and CD86, it could be selected as the source cell. Targeting peptides expressing plasmids (e.g., RVG (rabies virus glycoprotein)) were transfected into the source cells to get exosomes with the ability of specifically binding to neural cells. Therapeutic nucleic acids can be introduced into the modified exosomes by electroporation method. Following intravenous injection, exosomes encapsulating nucleic acids could be found within the central nervous system.

Exosomes serve as not only vehicles to deliver miRNA but also synthetic nucleic acids, for instance, short interfering RNA (siRNA) which are therapeutic compounds and have been exploited recently. Researchers obtained exosomes from self-derived dendritic cells and then decorated exosomes to express membrane protein Lamp2b and a neuron-specific RVG peptide in order to deliver cargoes specifically to recipient neural cells. Intravenously injected RVG-targeted exosomes delivered GAPDH siRNA which resulted in knockdown of BACE1 and mediated the formation of the peptide that forms *β*-amyloid plaque associated with AD pathogenesis [9]. These findings lighten the therapeutic potential of the application of exosome-mediated short interfering RNA approach in AD. Schematic representation of production, harvest, and readministration of targeted self-exosomes for gene delivery is illustrated in Figure 3.

By all indications, exosomes have multiple advantages over existing miRNA delivery vehicles. Since they are derived from the patients' own cells, they would be less immunogenic than other foreign delivery vehicles. Exosomes remain relatively stable in the blood as they avoid coagulation factors and complement which are most likely due to their surface expression of CD46 and CD59 [84], as well as antibody responses, due to their self-derived nature. Dendritic cell-derived exosomes express membranous tetraspanin CD9, which facilitates direct fusion with recipient cells and deliver their content directly into the cytosol [85]. This entry mode bypasses the endosomal-lysosomal pathway, where the immune system recognizes nucleic acids by TLR7 and TLR8. It also circumvents the need for endosomal-escape strategies with their associated toxicities. Finally, the small size of exosomes would also be beneficial with facilitation as they pass through vessel fenestrations.

Despite these advantages, numerous issues are still being addressed before clinical implementation of this strategy. Firstly, as to whether the RVG-Lamp2b fusion-protein induces adaptive immunity after repeated administration needs to be verified. In fact, adaptive immune responses would be anticipated. Another issue not yet addressed is that the receptor coupled with RVG expressed in the surface of exosomes, alpha-7-subunit acetylcholine receptor, markedly declines in brains affected by AD [10]. This issue is critical when aiming to develop RVG exosomes for clinical use in AD. In addition, a stable exosome source is yet to be exploited in order to expand well characterized exosomes. The loading process of miRNAs into exosomes also needs to be optimized to achieve maximum efficiency. Last but not the least, specific targeting function of exosomes should also be further studied to ensure safe and precise delivery to recipient tissues.

With this progress in intercellular transfer researches, exosomes provide a breakthrough in the field of drug delivery, bringing an alternative source of clinical therapy.

## 6. Perspectives and Conclusions

The ability of exosome-transporting miRNAs to function in neighboring or distant cells exhibits a novel insight into modification mechanisms employed in AD. Besides, our review also reveals gaps in knowledge that require further research.

As novel epigenetic-related systems ensure cellular information exchange, the exhaustive mechanism that underlies uptake of exosomes containing miRNAs into neighboring or distant cells needs to be explored and this holds enormous potential for RNA interference applications. In addition, investigating the ability of exosome-mediated miRNAs to bind to TLRs in the endosomes of recipient cells is still in its infancy. Vartanian and his team hypothesized that intracellular delivery of miRNAs via exosomes brings about TLR tolerance, which is a hyporesponsive state of TLR contributing to neuroprotection [86]. If this is the case, it reveals an exciting new insight into the regulation of TLRs within inflammation of AD and even in other CNS inflammation-related diseases. Overall, these compelling evidences that we summarize in this review point out a complex interplay between neurodegeneration and exosomal miRNAs and these support the functional effects of exosome-containing miRNAs in neurodegenerative disorders.

Although previous researches have demonstrated that a panel of miRNAs within exosomes from the CSF and plasma may serve as fingerprints of early stages in AD [3, 4, 6], further research data is still critical to identify the function of the miRNA in order to strengthen such findings. The diagnostic validation of targets (mRNA and protein) need to be coupled with miRNAs; therefore, one of the major challenges would be the comparison of miRNA analysis with transcriptions and proteomic studies. Besides, technological aspects possessing both considerable production and meticulous analysis represent another challenge. With the need to further understand miRNA function in specific cellular events, consequent technological advances in sensitive and accurate miRNA analysis are necessary for offering prospective role for exosomal miRNAs to be used as potential diagnostic biomarkers of AD.

A study that demonstrates an efficacious way of delivering functional siRNA into AD mouse brain by systemic exosome administration represents a promising approach for RNA interference applications [9, 10]. However, we have to realize that there is still a long way ahead, and numerous hurdles need to be crossed. First of all, since the possibility of potential side effects on recipient cells has not been looked at, the safety of exosomes should never be ignored. Moreover, the precise mechanisms that underlie the processes of the blood-brain barrier cross, target cells selection, and content release have not been proven clearly, and this necessitates further investigation. Further researches are needed to establish if exosomal miRNAs could be utilized as diagnostic biomarkers of AD and practical RNA interference applications.

This review has summarized compelling evidence for multiple ways in which exosomal miRNAs contribute to important biological functions related to intercellular communication, neuroinflammation, and neuroplasticity. We put forward new applications of exosomal miRNAs as diagnostic biomarkers and potential gene therapy. We expect that our review will contribute to better comprehension of the roles that exosomal miRNAs play in pathological mechanisms of AD.

## Figures and Tables

**Table 1:** Epigenetically regulated exosomal microRNAs linked to neurodegeneration.

MircoRNA	Role in neural network	Association with neurodegeneration	Biological fluids
EBV-miRNA	Intercellular communication	Gene silencing	Cerebrospinal fluid
miRNA-124a	Intercellular communication	Neuron-to-astrocyte signaling	Cerebrospinal fluid
miRNA-219	Neuronal differentiation and development	Promote CNS myelination	Cerebrospinal fluid, plasma
miRNA-133b	Neuronal plasticity	Benefit neurite remodeling	Cerebrospinal fluid
miRNA-193b	Neuronal differentiation	Affect A*β* generation	Cerebrospinal fluid, plasma
miRNA-101	Neuronal differentiation	Affect A*β* generation	Cerebrospinal fluid
miRNA-29c	Neuronal differentiation	Affect A*β* generation	Cerebrospinal fluid
miRNA-33	Neuronal maturation and apoptosis	Downregulation influence ApoE lipidation and A*β* metabolism	Cerebrospinal fluid
miRNA-195	Neuronal differentiation	Deregulate amyloid metabolism and tau phosphorylation	Cerebrospinal fluid
let-7	Neuronal inflammation	Activate the RNA-sensing TLR7	Cerebrospinal fluid
miRNA-146a	Neuronal inflammation	Modulate microglial function	Cerebrospinal fluid
